# Supplementary material for: Temporal Characterization of the Amyloidogenic APPswe/PS1dE9;hAPOE4 Mouse Model of Alzheimer’s Disease
Source: Int J Mol Sci. 2024 May 25;25(11):5754. doi: 10.3390/ijms25115754 (PMC11172317; doi:10.3390/ijms25115754)
Supplement: Supplementary file 1 [file ijms-25-05754-s001.zip › ijms-3019344-supplementary.pdf]

International Journal of
Molecular Sciences

MDPI

Table S1. Mouse *N* values. Overview of mice utilized per genotype, broken down by age and sex.

	**APPPS1:E4**			**APPPS1:E3**		
**Age**	**8**	**12**	**16**	**8**	**12**	**16**
**Male *(N)***	4	4	7	2	1	2
**Female *(N)***	5	3	4	2	2	1
**Total *(N)***	9	7	11	4	3	3

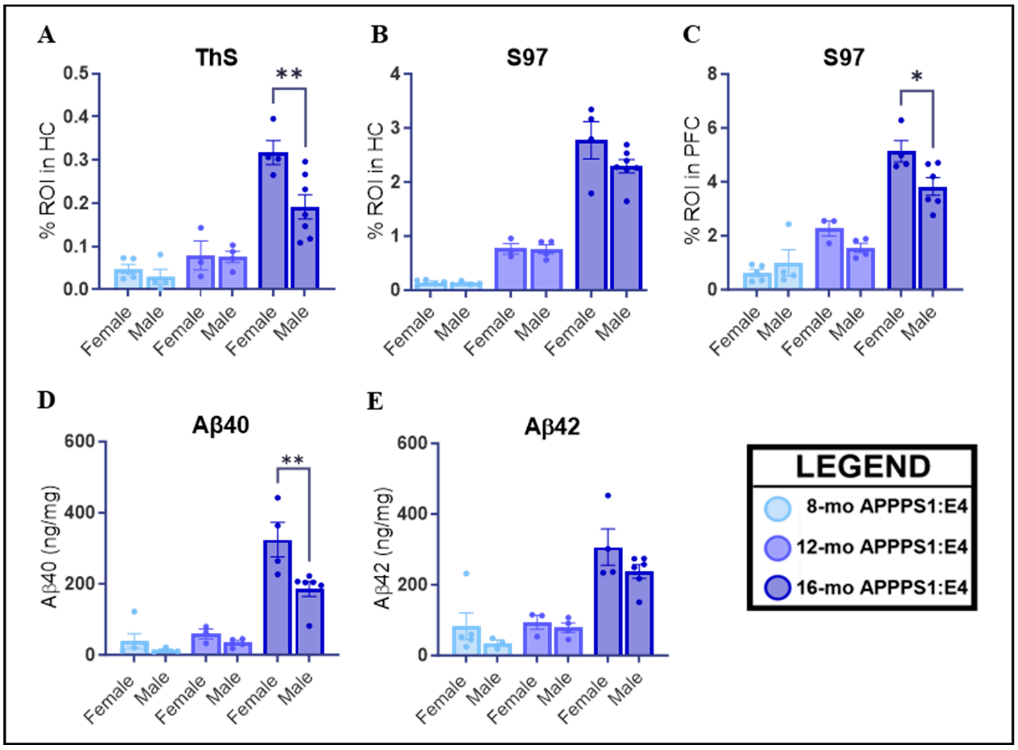

Figure S1: Sex differences in general Aβ deposition. Sex differences in Aβ deposition in the hippocampus and prefrontal cortex of APPPS1:E4 mice. Data are presented as mean ± SEM. Sex effect was analyzed with two-way ANOVA followed by post hoc Sidak's correction (simple effects within age). * $p < 0.05$; ** $p < 0.01$.

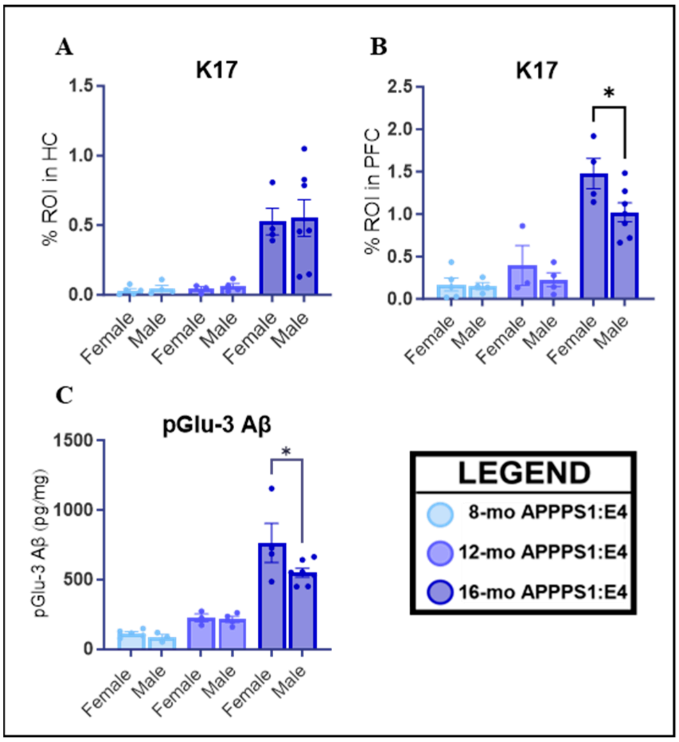

Figure S2: Sex differences in in pGlu3-Aβ deposition. Sex differences in pGlu3-Aβ deposition in the hippocampus and prefrontal cortex of APPPS1:E4. Data are presented as mean ± SEM. Sex effect was analyzed with 2-way ANOVA followed by post hoc Sidak's correction (simple effects within age). * $p < 0.05$.

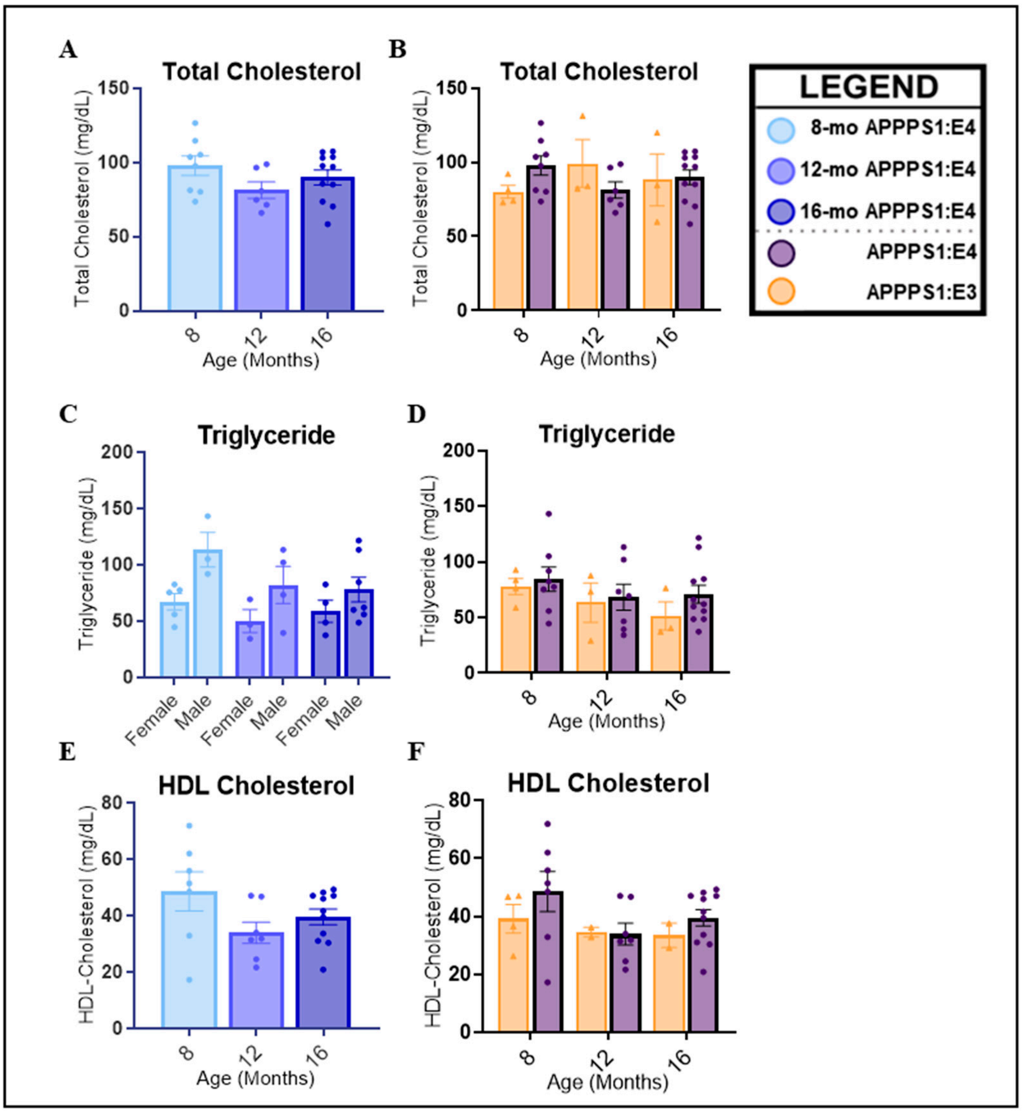

Figure S3: Blood plasma lipid levels. Colorimetric analysis of **(A,B)** total cholesterol **(C,D)** HDL cholesterol and **(E,F)** triglycerides in the blood plasma of APPPS1:E4 and APPPS1:E3 mice. Data are presented as mean ± SEM. APP/E4 age effect analyzed with 2-way ANOVA followed by Tukey's HSD post-test (simple effects within sex). With sexes pooled, genotype effect was analyzed by two-way ANOVA followed by post hoc Sidak's correction (simple effects within age).

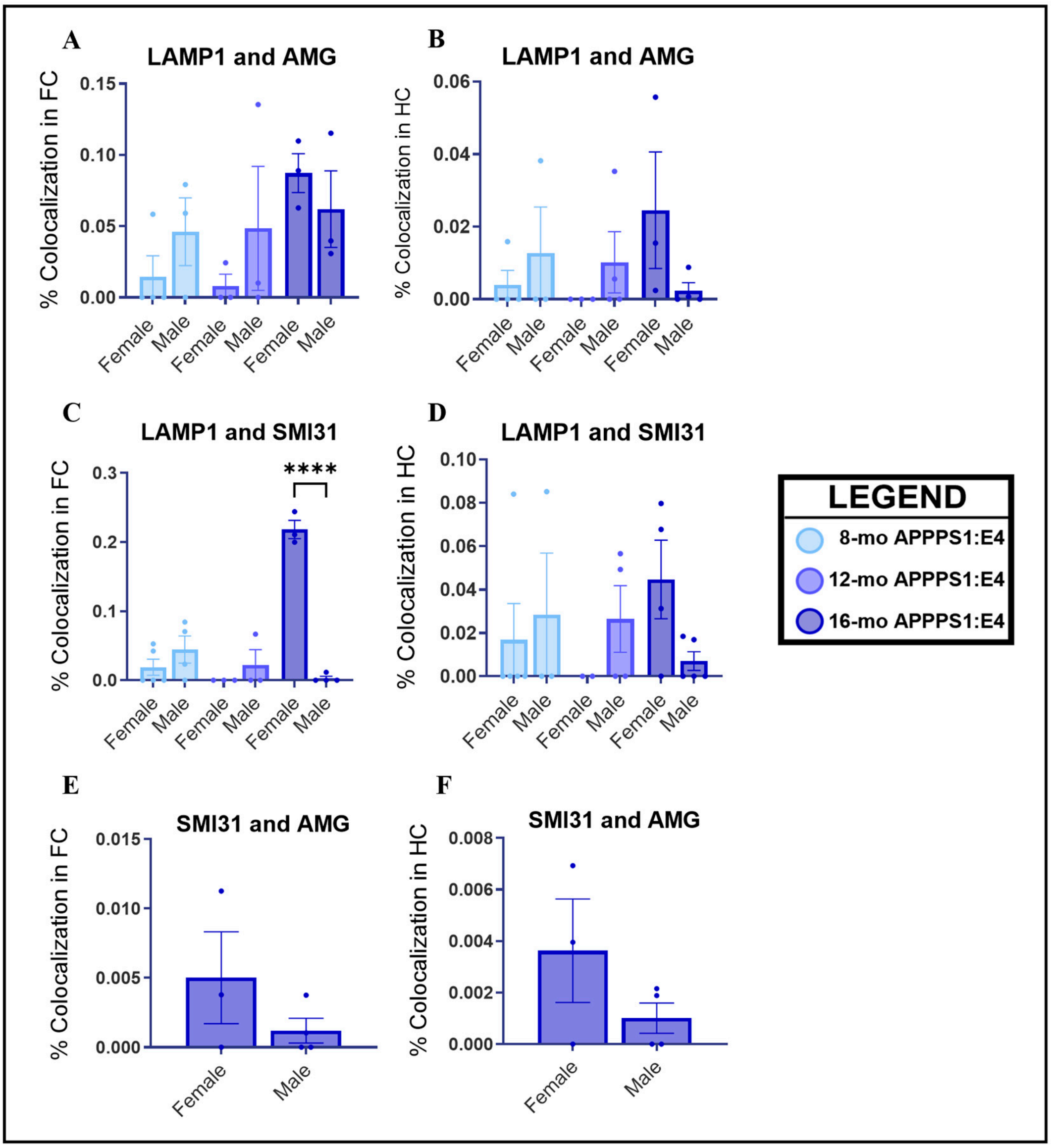

Figure S4: Sex differences in lysosomal dysfunction & neuritic dystrophy. Sex differences in lysosomal dysfunction & neuritic dystrophy in the hippocampus and frontal cortex of APPPS1:E4 mice. Data are presented as mean ± SEM. Colocalization data are presented in arbitrary units. Sex effect was analyzed by two-way ANOVA followed by post hoc Sidak's correction (**A, B, C, D**) or by Welch's test (**E, F**). **** $p < 0.0001$. HC: hippocampus; FC: frontal cortex; AMG: Amylo-Glo.
